# The inhibitory effect of melatonin on human prostate cancer

**DOI:** 10.1186/s12964-021-00723-0

**Published:** 2021-03-15

**Authors:** Dexin Shen, Lingao Ju, Fenfang Zhou, Mengxue Yu, Haoli Ma, Yi Zhang, Tongzu Liu, Yu Xiao, Xinghuan Wang, Kaiyu Qian

**Affiliations:** 1 Department of Urology, Zhongnan Hospital of Wuhan University, Wuhan, China; 2 Department of Biological Repositories, Zhongnan Hospital of Wuhan University, Wuhan, China; 3 Human Genetics Resource Preservation Center of Hubei Province, Wuhan, China; 4 Wuhan Research Center for Infectious Diseases and Cancer, Chinese Academy of Medical Sciences, Wuhan, China; 5 Cancer Precision Diagnosis and Treatment and Translational Medicine, Hubei Engineering Research Center, Wuhan, China; 6 Emergency Center, Zhongnan Hospital of Wuhan University, Wuhan, China; 7 Center for Life Sciences, Peking University, Beijing, China; 8 Peking-Tsinghua Center of Life Sciences, Beijing, China; 9 Euler Technology, ZGC Life Sciences Park, Beijing, China; 10 Medical Research Institute, Wuhan University, Wuhan, China

**Keywords:** Prostate cancer, Melatonin, AR, ADT, P27^Kip1^

## Abstract

Prostate cancer (PCa) is one of the most commonly diagnosed human cancers in males. Nearly 191,930 new cases and 33,330 new deaths of PCa are estimated in 2020. Androgen and androgen receptor pathways played essential roles in the pathogenesis of PCa. Androgen depletion therapy is the most used therapies for primary PCa patients. However, due to the high relapse and mortality of PCa, developing novel noninvasive therapies have become the focus of research. Melatonin is an indole-like neurohormone mainly produced in the human pineal gland with a prominent anti-oxidant property. The anti-tumor ability of melatonin has been substantially confirmed and several related articles have also reported the inhibitory effect of melatonin on PCa, while reviews of this inhibitory effect of melatonin on PCa in recent 10 years are absent. Therefore, we systematically discuss the relationship between melatonin disruption and the risk of PCa, the mechanism of how melatonin inhibited PCa, and the synergistic benefits of melatonin and other drugs to summarize current understandings about the function of melatonin in suppressing human prostate cancer. We also raise several unsolved issues that need to be resolved to translate currently non-clinical trials of melatonin for clinic use. We hope this literature review could provide a solid theoretical basis for the future utilization of melatonin in preventing, diagnosing and treating human prostate cancer.

**Video abstract**

**Supplementary Information:**

The online version contains supplementary material available at 10.1186/s12964-021-00723-0.

## Background

### Prostate cancer

Prostate cancer (PCa) is one of the most commonly diagnosed cancers in males [1]. The American Cancer Society estimated 191,930 new cases and 33,330 new deaths of PCa in 2020 [1]. Sadly, although there is a downward trend in the number of new cases and deaths for several decades [1], conditions seem to deteriorate during the past 3 years (2017–2019) [2–4]. The estimated new cases per year increased from 161,360 in 2017 [2] to 174,650 in 2019 [4] and the annual death increased from 26,730 in 2017 [2] to 31,620 in 2019 [4]. Androgen and androgen receptor (AR) pathways played essential roles in the pathogenesis of PCa [5]. Aberrant AR mutations [6], intratumoral androgen synthesis [7], and abnormal AR splice variants [8] are negative inducers of PCa. Currently, the most used therapy for hormone-naive PCa cancer patients is androgen depletion therapy (ADT). ADT could lead patients to an initially beneficial stage with tumor extinction, but eventually the majority of hormone-naive PCa become less sensitive to ADT and progress into a more malignant stage, the castration resistant prostate cancer (CRPC). CRPC not only is resistant to castration treatment but also exhibits other negative features such as distant metastasis [9] and neuroendocrine differentiation. Due to the high relapse and mortality of PCa, developing novel noninvasive therapies have become the focus of research. Among the artificial and natural compounds, melatonin is of great potential for treating PCa.

### Melatonin

Melatonin (N-acetyl-methoxy-tryptamine), an indole-like neurohormone, is primarily synthesized in human pineal gland. Besides brain, melatonin is also found to be synthesized in lung [10], gastrointestinal tract [11], lens [12], kidney [13] and pancreas [14]. Daily circadian rhythm is the main regulator that modulates the biosynthesis of melatonin [15]. Optical signals promote the breakdown of melanopsin in retinal photoreceptive ganglion cells, thus blocking the synthesis of melatonin [16]. Consequently, melatonin in circulation is low or even undetectable during the daytime [17]. On the contrary, melatonin is mainly produced during the dark phase, and the maximal plasma level is detected 4–5 h after darkness onset [18]. Alternating light–dark cycles or improper exposure to light can disrupt the inner circadian rhythm and thus decrease nocturnal melatonin synthesis [19, 20]. The most prominent function of melatonin is its high efficiency as a radical scavenger [21]. The amphiphilic nature allows melatonin to easily enter mitochondrion [22–24]. Through scavenging free radicals, activating uncoupling proteins and maintaining mitochondrion membrane potential, melatonin could effectively maintain intracellular redox homeostasis [25, 26]. Recently, mitochondrion is pointed out to be the potential site where melatonin is originally generated [27]. The other bio-functions of melatonin included but not limited to regulating metabolism [28], promoting vascular reactivity [29], elevating immune response [10], preventing DNA damage [30], inducing apoptosis [31] and autophagy [32–34] and inhibiting tumor proliferation [34–36].

### The intake of melatonin from daily diet or drug supplements

Daily diet can provide a certain amount of melatonin for human body ranging from picograms to milligrams per gram. For foods of animal origin, eggs, fish and milk contained a higher concentration of melatonin [37]. Interestingly, milking time was a key factor that determined the concentration of melatonin in milk [38]. According to Milagres et.al [39], milk produced at night contained a relatively higher concentration of melatonin than milk produced during the daytime. For foods of plant or fruit sources, the generative organs tended to contain a higher degree of melatonin, especially the seeds, which were proposed as having a considerably higher concentration of melatonin than other vegetable tissues [40]. In a study including 12 healthy male volunteers, the volunteers were asked to drink juice extracted from 1 kg of orange or pineapple or two whole bananas for breakfast and the serum melatonin concentration reached the highest level at the second hour after breakfast [41]. Moreover, serum antioxidant capacity of the volunteers was also obviously improved and was significantly correlated with serum melatonin concentration for all the three fruits tested.

Moreover, melatonin can also be obtained from drug supplements. In Europe and the United States, melatonin-supplement drugs were wildly used to alleviate jet-lag or to improve sleep quality. Noteworthy, traditional Chinese medicine also contained a degree of melatonin ranging from a few to several thousand nanograms per gram, revealing that they were ideal natural origins of this health-friendly hormone [42].

In a word, people’s daily diet or commercial melatonin-supplement drugs are good sources of melatonin and a reasonable combination of food ingredients could bring benefits to human body.

### Objectives

Considering that the number of reviews of the inhibitory effect of melatonin on PCa in recent 10 years is not commensurate with the advances acquired in relevant fields, we write this literature review to fill this gap. Herein, we systematically discuss the anti-PCa property of melatonin to summarize current understandings about its inhibitory effect on human PCa and uncover the possibility of translating experimental findings into clinical treatment.

## The inhibitory property of melatonin

### The correlation between melatonin disruption and risk of PCa

As described above, the release of melatonin is under the control of circadian rhythm and light exposure. Disturbed circadian rhythm of light and decreased serum level of melatonin is highly correlated to various types of human cancers [43–47]. Notably, the incidence of cancer in blind people is surprisingly lower than in normal people without visual impairment [48–51], which may be a result of a higher level of melatonin. In 2007, the World Health Organization once concluded that “shift-work that involves circadian disruption is probably carcinogenic to humans” [52].

Actually, the potential correlation between melatonin and the risk of PCa has arisen people’s attention as early as 1985. A study conducted in healthy young men, BPH patients and PCa patients showed that the serum level of melatonin waves the most dramatically compared with other hormones, such as prolactin or growth hormone, reminding people of an unexpected association between melatonin and human PCa [53].

Recently, epidemiologic surveys have exhibited that disrupted secretion and low blood levels of melatonin may elevate the risk of PCa in humans (Table 1). According to Papantoniou et al. [54], they discovered a higher incidence of PCa among people who had worked at least for 1 year in night shift work. Moreover, in a 1693-people-involved study, Wendeu-Foyet et al. [55] reported that male workers who underwent permanent night work for at least 20 years were more inclined to suffer from advanced PCa. In addition, along with cortisol, another hormone that is related to the circadian cycle, Tai et al. [56] detected a higher level of PSA while a lower level of aMT6s (a metabolite of melatonin) in PCa patients than healthy men. Besides night shift work, improper exposure to light may also be responsible for the initiation of PCa. A case–control study in South Korea [57] found light pollution is an independent risk factor of PCa. However, supplementing melatonin in nocturnal drinking water could recover melatonin to a normal level and reverse these negative effects [43].

**Table 1:** The correlation of melatonin disruption and risk of prostate cancer

Research object	Study type	Results	References
818 incident PCa cases and 875 frequency-matched controls in France	Case–control study	A long duration of at least 20 years of permanent night work was associated with aggressive PCa (OR 1.76, 95% CI 1.13, 2.75), even more pronounced in combination with a shift length > 10 h or ≥ 6 consecutive nights (OR 4.64, CI 1.78, 12.13; OR 2.43, CI 1.32, 4.47, respectively)	Wendeu-Foyet et al. [54]
120 newly diagnosed PCa patients and 240 age-matched controls in Taiwan, China	Case–control study	Compared with patients with lower urinary melatonin-sulfate or melatonin-sulfate/cortisol (MT/C) ratio levels, those with above-median levels were significantly less likely to have PCa (adjusted OR 0.59, 95% CI 0.35–0.99; adjusted OR 0.46, 95% CI 0.27–0.77) or advanced stage prostate cancer (adjusted OR 0.49, 95% CI 0.26–0.89; adjusted OR 0.33, 95% CI 0.17–0.62)	Tai et al. [55]
Aim to elucidate the association between ALAN and PCa in 27 districts within Gwangju City and urban and rural areas from South Jeolla Province in South Korea	Case–control study	The incidence of PCa was significantly associated with ALAN (risk ratio 1.02, p 0.0369), no significant association was observed between ALAN and other cancers	Kim et al. [56]
111 men with PCa, 928 men without PCa in Iceland	Case–cohort study	Men with morning aMT6s levels below the median had a fourfold statistically significant increased risk for advanced PCa compared with men with levels above the median (hazard ratio: 4.04; 95% CI 1.26–12.9)	Sigurdardottir et al. [45]
1,095 PCa cases and 1,388 randomly selected population controls in Spain	Case–control study	Subjects who had worked at least for 1 year in night shift work had a slightly higher PCa risk (OR 1.14; 95% CI 0.94, 1.37) compared with never night workers; OR increased with longer duration of exposure (≥ 28 years: OR 1.37; 95% CI 1.05, 1.81; p-trend = 0.047)	Papantoniou et al. [53]
Male, pigmented, homozygous, athymic, inbred nude rats	Experimental study	Rats in blue-tinted cages evinced over sixfold higher peak plasma melatonin levels at mid-dark phase and the amplification of nighttime melatonin levels by exposing nude rats to blue light during the daytime significantly reduces human PCa metabolic, signaling, and proliferative activities	Dauchy et al. [58]

*OR* odds ratio, *CI* confidence interval, *ALAN* artificial light at night

**Table 2:** The synergistic use of melatonin and other drugs or therapies in treating prostate cancer

Synergistic use	Subject	Results	References
Melatonin and castration therapy	androgen-sensitive PCa cells	Melatonin significantly slowed the appearance and growth rate combined with castration therapy compared with the untreated group	Siu et al. [101]
Melatonin and triptorelin	CRPC patients	The concomitant administration of the melatonin with castration therapy in CRPC patients could induce an obvious decrease in PSA serum levels, elevate platelet count to a standard value and prolong the overall survival span	Lissoni et al. [105]
Melatonin and hormone radiation treatment	PCa patients	Long-term use of melatonin was an independent predictive factor and could reduce the event of death to lower than 50% in PCa patients with advanced stage	Gennady et al. [106]
Melatonin and DHA	PCa cells	Melatonin combined with DHA could suppress proliferative prostate diseases through modulating mitochondrion bioenergy via AKT and ERK1/2 pathway	Tamarindo et al. [208]
Melatonin and cryopass-laser treatment	LNCaP cells and 7-week old Foxn1 ^nu/nu^ mice	Melatonin could be precisely delivered to specific areas avoiding false distribution in non-target tissues and unwanted side-effects via cryopass-laser and 3 mg/kg/week melatonin could effectively inhibit the proliferation of LNCaP PCa cells	Terraneo et al. [209]

The underlying explanation might be that light at night could suppress melatonin biosynthesis and damage normal metabolism thus activating aberrant proliferative activity [58]. While changing the wavelength of light to a proper range, for example, blue light could amplify the nighttime melatonin level and inhibit the metabolism and proliferative activities of PCa cells [59] (Table 2).

Inspiringly, due to the significance of personalized PCa screening has been increasingly prominent, traditional PSA screening strategies are urged to be developed [60] and considering the serum level of melatonin or the level of its metabolite is closely related to the risk of PCa, it deserves further exploring that whether the combined detection of PSA and melatonin can further improve the efficiency of existing PCa screening methods.

## Melatonin and androgen receptor (AR) pathway

### The AR pathway

AR pathway, including androgen, AR and AR co-regulators, is of primary significance in the biogenesis and development of PCa [61–63]. Androgen synthesized in testis hold nearly 60% of the gross in prostate gland [64]. The rest 30% is mainly secreted from the zona reticularis of adrenal glands [65, 66]. Androgen acts as a ligand in this pathway and activates downstream signals by binding to androgen receptor (AR), which is also known as a nuclear transcription factor. AR has four domains: the C-terminal ligand-binding domain (LBD); the centrally-located DNA-binding domain (DBD); the N-terminal transactivational domain (NTD) and the hinge region that separates the LBD and the DBD. The interaction between LBD and NTD is essential for AR to maintain its stability. Unliganded AR mainly resided in the cytoplasm [67, 68] remaining an inactive status being sequestered by multiple heat shock proteins or cytoskeletal proteins [69]. After being coupled with androgen, the complex is triggered to dissociate and the following interaction between AR and Filamin A promotes the nuclear translocation of AR. Then AR binds to specific DNA sequence motifs, the androgen response elements (AREs), in the promoter and enhancer region of these hormone-dependent genes to initiate transcription.

#### The role of AR in PCa

The role of AR in driving the initiation and progression of PCa has been well established. More concretely, AR participants in the development and drug-resistance of PCa mainly via three routines. The first one is aberrant AR mutations. Mutations in the LBD domain of AR, such as residue 701 (L701H) and residue 877 (T877A), are related to distant metastasis [70] and abnormal activation of AR [71, 72]. Another three mutations, L702H, W742C and H875Y, are identified to be associated with the development of CRPC [73, 74]. The second is intratumoral androgen synthesis. Although castration can significantly reduce circulating testosterone levels, the total amount of serum androgen metabolites and androgen isolated from PCa tissues still have pathological effects, indicating a non-testicular source of androgen [65, 75]. Indeed, CYP11A1, a cholesterol cleavage enzyme, is overexpressed in CRPC tissues and participants in the process of a weak androgen synthesis [7]. Last but not least is the abnormal expression of AR and AR splice variants (AR-Vs). The aberrant amplification of AR could hypersensitize PCa cells to a low level of androgen [76] and cause resistance to anti-androgen drugs like bicalutamide [77]. AR-Vs, including AR-V1, AR-V567es, and AR-V7, are truncated forms of AR protein lacking the LBD [78] and have frequently been detected in CRPC tissues. AR-V7 is the most thoroughly explored one due to its abundance and is currently utilized as a clinical biomarker for therapy selection in men with distant metastasis [79]. Due to the loss of LBD, AR-Vs can escape the regulation of current hormone therapies [80]. Interestingly, since they retain the DBD, AR-Vs are still capable of regulating the transcription of downstream genes and further promote the occurrence of CRPC [80].

#### The adverse effect of melatonin on AR pathway

Melatonin has been demonstrated to modulate the transcription activity of the estrogen receptor (ER) and inhibit the expression of the estradiol-dependent gene [81, 82]. However, melatonin does not damage the activation of AR by androgen. AR mainly works in the nuclear and the right localization of AR is indispensible for its biological activity. Nuclear exclusion is caused by mutations in the DBD of AR and results in loss of androgen sensitivity [83]. Moreover, the mislocation of AR is confirmed to promote human diseases such as spinal bulbar muscular atrophy (SBMA) [84, 85].

**Fig. 1:**
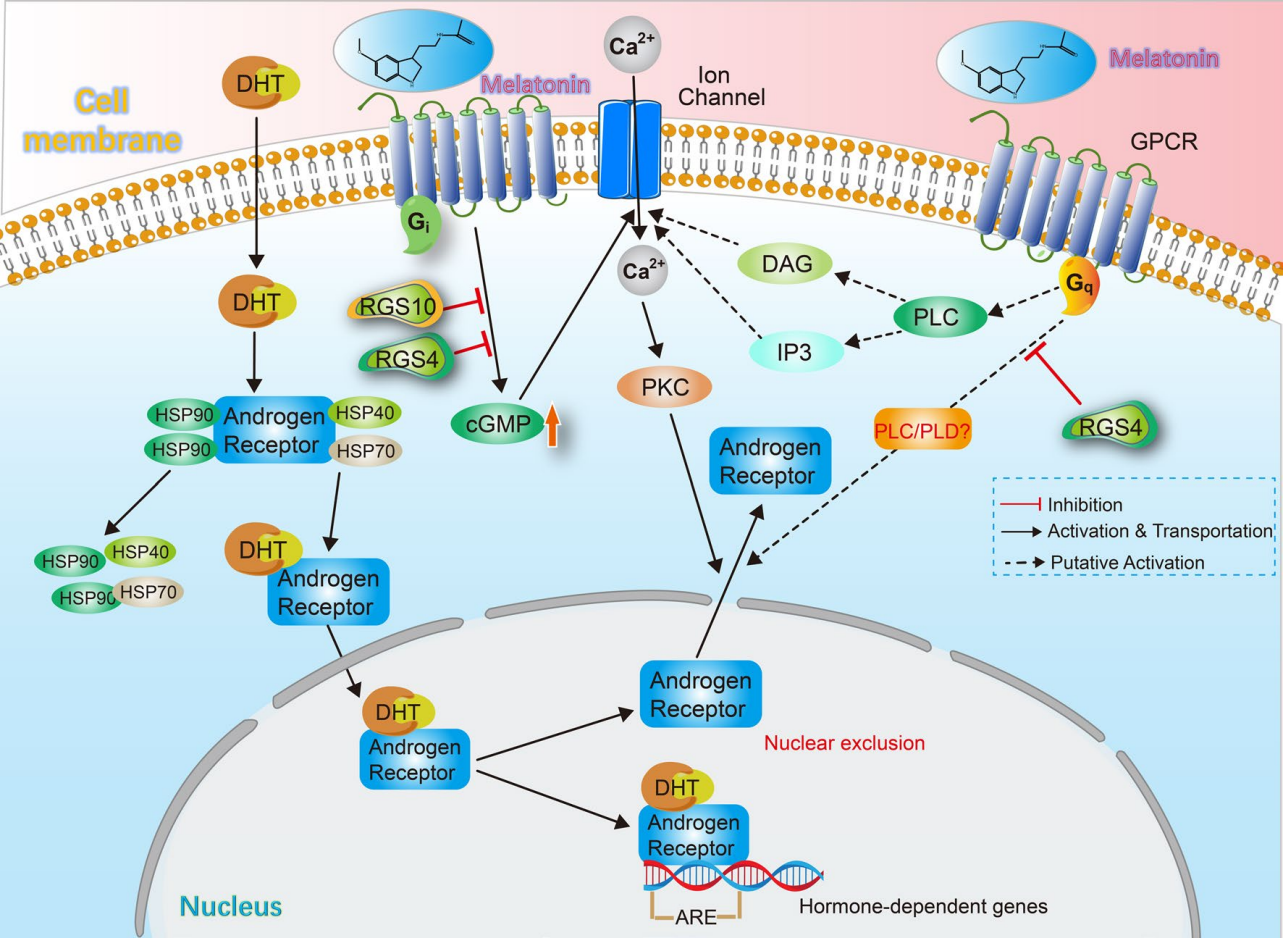
Adverse effect of melatonin on the AR pathway. (1) The AR pathway. Androgen triggered the dissociation of AR and HSPs. AR and androgen formed complexes and were transported into nuclear. Nuclear AR complexes bound to the ARE region of hormone-dependent genes to start the transcription. (2) Melatonin mediated the AR nuclear exclusion via a G_i_/cGMP/Ca^2+^/PKC pathway. Melatonin stimulated G_i_ protein to enhance the production of cGMP. cGMP induced the intracellular flux of Ca^2+^. Ca^2+^ activated PKC and PKC promoted the nuclear exclusion of AR. RGS proteins (RGS4, RGS10) exerted adverse effects on this pathway. (3) The potential involvement of G_q_ protein in AR nuclear exclusion. Melatonin activated phospholipase C (PLC) to generate inositoltriphosphate (IP3) and diacylglycerol (DAG) which contributed to the intracellular flux of Ca^2+^. Activated G_q_ protein presumably stimulated PLC or (PLD) to promote AR nuclear exclusion, and the process was inhibited by RGS4

Rimler et al. [86] showed that melatonin treatment could increase the protein level of AR but not inhibit its binding capacity as a transcription factor. Furthermore, although the overall amount of AR in the cells was elevated, AR content present in nuclear was unexpectedly reduced. Another two studies by Rimler et al. [87, 88] also confirmed that the regulation of melatonin on AR is mainly via promoting its nuclear translocation rather than blocking its expression level or competing the steroid binding sites. According to Lupowitz et al. [89] and Rimler et al. [90], the binding of melatonin and its receptor stimulated Gi-type G proteins to enhance the production of cGMP. Elevated cGMP induces intracellular flux of Ca^2+^ with the following activation of PKC. Activated PKC finally promoted the exclusion of AR with an unclarified Gq-signaling pathway. Additionally, the concomitant activity of RGS proteins exerts adverse effects on the melatonin-triggered AR exclusion. The mechanism diagram of AR nuclear exclusion is shown in Fig. 1.

The findings presented above contribute to a better understanding of the androgen inhibitor action of melatonin and provide solid theories for exploring new AR localization regulators to improve the drug sensitivity of PCa.

#### The benefits of melatonin on androgen depletion therapy

Because of the primary role of AR pathway in fueling the initiation and growth of PCa, androgen depletion therapy (ADT) or castration therapy has become the first-line treatment for PCa. Castration can be performed via two methods, bilateral orchiectomy or chemical drugs, to reduce the serum testosterone to an extremely low level (≤ 50 ng/dL) [91]. However, ADT is only a palliative therapy but not a curative therapy for PCa p atients. Although there is a positive feedback rate of 80–90% in the early stage of treatment [92], the majority of patients will finally transform into the stage of CRPC, a more aggressive and lethal stage of PCa. The average overall survival of CRPC patients with distant metastasis is 1.5 years [93].

Although the level of androgen in circulation is extremely low, a number of studies still show that androgen and AR pathway remain functional in CRPC cells. A gene expression analysis of PCa samples during hormonal therapy unexpectedly revealed that the overall expression patterns of CRPC were nearly identical to those of the untreated samples [94]. For example, FKBP5, a hormone-responsive gene regulated by AR, is identified to be re-expressed in CRPC samples even though under an androgen-depleted condition [94, 95]. Moreover, studies inhibiting AR expression surprisingly showed that AR is indispensable for PCa cells to maintain the growth in vitro and in castrated mice [96–99]. In general, CRPC is not totally hormone refractory and the AR pathway remained an important therapeutic target for CRPC [100].

**Fig. 2:**
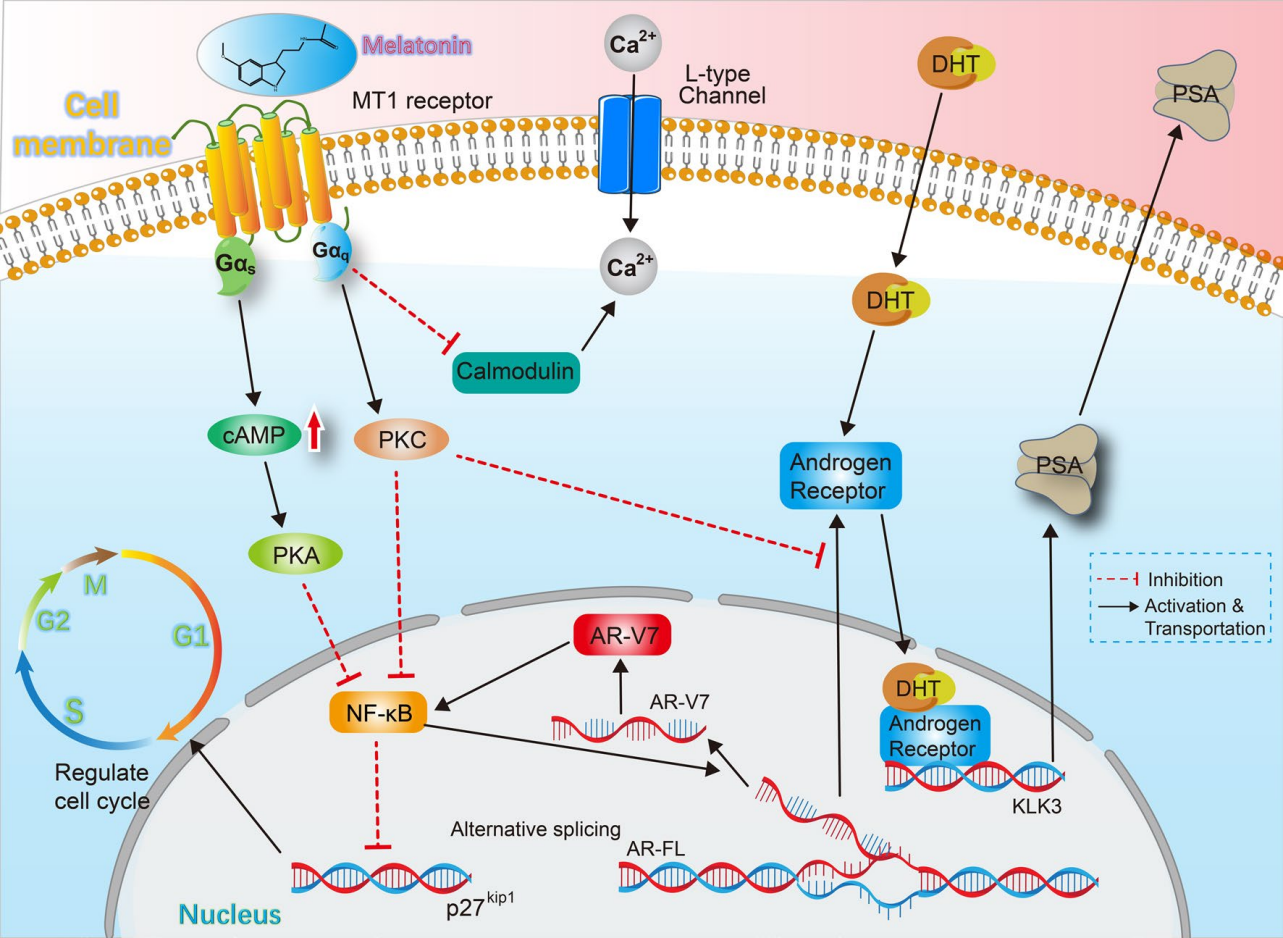
The MT1 pathway. (1) Melatonin up-regulated p27^kip1^ via blocking the activity of NF-κB. MT1 receptor activated Gαs and Gαq. Gαq directly activated PKC and Gαs indirectly activated PKA via elevating cAMP. PKC and PKA inhibited the binding ability of NF-κB to the promoter region of p27^Kip1^ gene. (2) Melatonin decreased the PSA level with an attenuated Ca^2+^ influx. Activated PKC inhibited the promoting effect of DHT and AR on KLK3 (PSA gene). MT1 receptor inhibited melatonin-responsive calmodulin to modulate L-type Ca^2+^ channel. (3) Melatonin interrupted the bi-directional positive interactions between AR-V7 and NF-κB. The MT 1 receptor-mediated inhibition on NF-κB decreased the formation of AR-V7 and thus blocking the interactions between AR-V7 and NF-κB

Notably, some studies have shown positive outcomes by adding melatonin to ADT. Siu et al. [101] reported that melatonin can strengthen the inhibitory effect of castration therapy on androgen-sensitive PCa cells. By establishing a castrated-model, they found melatonin significantly slowed the appearance and growth rate combined with castration therapy compared with the untreated group. Along with their previous observations, melatonin could inhibit the proliferation of LNCaP cells both in vitro under an androgen-free condition [102] and in intact mice [103]. These data supported an ideal synergistic benefit of melatonin and castration in clinical use. Besides androgen-sensitive prostate cancers, melatonin also exerts a positive effect on hormone-refractory PCa cells. Liu et al. [104] reported that melatonin can delay the progression of castration resistance in advanced PCa via interrupting the reciprocal interaction between AR-V7 and NF-κB (shown in Fig. 2). Moreover, the concomitant administration of the melatonin with castration therapy in CRPC patients could induce an obvious decrease in PSA serum levels, elevate platelet count to a standard value and prolong the overall survival span [105]. This pilot study demonstrated the feasibility of melatonin repletion to overcome the loss of efficacy of castration therapy and improve the clinical effect for advanced PCa patients. Moreover, as Gennady et al. [106] reported, long-term use of melatonin was an independent predictive factor and could reduce the event of death to lower than 50% in PCa patients with advanced-stage, even though it did not show ideal effects on patients with a favorable and intermediate prognosis.

“The treatment of castration-resistant prostate cancer (CRPC) has entered a renaissance era” [107] and considering the fact that the majority of patients will recur and develop into an advanced stage although with an early effective period, there is an urgent need for original, efficacious, secure, and affordable therapies [108, 109]. The results described above highlights the bright future of synergistic interactions between melatonin and ADT in clinical utilization.

### Melatonin and MT1 signaling pathway

Generally, it is well-established that there exist two classical types of melatonin receptors in cells, the nuclear RZR/ROR receptors and the membrane-bound MT1 receptors and MT2 receptors. The nuclear RZR/ROR receptors are the relatively less examined ones that are reported to function in the nucleus. The MT1 receptors and MT2 receptors belong to a small sub-family of the GPCR superfamily [110–112] and these two membrane-bound receptors trigger most of the signaling pathways of melatonin. The signaling pathways activated by melatonin receptors here we discuss are dependent on tissue context [113, 114] which made it hard to attribute the anti-cancer ability of melatonin to one specific receptor. For example, it was firstly believed that melatonin induces cell apoptosis in colon tumors mainly through the nuclear RZR/ROR receptors [115, 116], while later reports showed that the MT2 receptors also mediated the anti-tumor property on intestinal cancers [116, 117]. And in breast cancers, melatonin was reported to exert its oncostatic action mainly via its MT1 receptors [118, 119].

In PCa, reports showed that MT1 receptors mediated the anti-tumor events of melatonin. Xi et al. [102] reported that melatonin at a pharmacological concentration could inhibit LNCaP cell growth via MT1 receptors with an attenuated Ca^2+^ influx and the consequent decrease in the level of PSA in the supernatant fluids. Moreover, in another study, Xi et al. [103] also reported that besides causing retarded proliferation of LNCaP cells xenograft in nude mice, mean decreases of 51.7% and 38.7% in tumor volumes were recorded in mice given daily melatonin injection initiated 10 days pre- and post-tumor cell transplantation, respectively. Moreover, cell proliferation markers, the PCNA and cyclin A, decreased obviously after melatonin treatment in LNCaP cells. Besides using standard cell lines, a proof-of-concept translational study using clinical samples showed clear evidence that MT1 signaling is crucial for melatonin to exert its anti-tumor function [120]. Immunohistochemistry assays demonstrated that the receptor subtype of the hormone-refractory patient was identical to the MT1 receptors in PC3 cells. And clinical observations showed that 5 mg melatonin per day not only stabilized the PSA level for almost 6 weeks but also retarded the biological development of the disease with a 23% increase in PSA double time and the platelet count is maintained at a relatively low-risk level.

To date, although none specific genes or chromosomal regions have been indicated to be responsible for PCa initiation, the loss of p27^Kip1^ was proved to have a major significance in the initiation and maintenance of PCa [121, 122]. p27^Kip1^ is one of the best-known tumor suppressors which functions as a cyclin-dependent kinase inhibitor preventing cells from entering the G1 phase. Several reports have confirmed that p27^Kip1^ expression level is adversely associated with the prognosis of PCa [123, 124]. Recently, a series of studies demonstrated the steps of how melatonin up-regulates the p27^Kip1^ expression [125–127]. Firstly, upon binding with melatonin, two G proteins, Gα_s_ and Gα_q_, were continuously activated. Then Gα_q_ directly activated PKC, while Gα_s_ indirectly activated PKA via increasing intracellular cAMP level. Next, the co-activated PKA and PKC inhibited the DNA binding ability of NF-κB to the promoter region of p27^Kip1^ gene, thus abolishing the repressing effect of NF-κB on p27^Kip1^. In addition, activated PKC was also capable of decreasing the PSA level by inhibiting the promoting effect of DHT mediated by AR. Thus, by directly up-regulating p27^Kip1^ and indirectly decreasing the PSA level (Fig. 2), melatonin exerted its latent capacity for preventing and treating prostate cancers.

### Melatonin and PCa metabolism

Tumorigenesis-associated metabolism, a key phenotype change during the oncogenic transformation, offers tumor cells survival opportunities to gain indispensable substances from a relatively nutrient-deficient environment. Most human solid tumors share the most common feature, the Warburg effect, markedly higher consumption of glucose compared with the surrounding normal tissue cells [128–130]. The prostate gland is a secretory organ that synthesizes and secrets metabolically distinct prostatic fluid containing a high concentration of citrate. Commonly, cells rely on citrate to proceed with the Krebs cycle for the progression of aerobic respiration and NADPH production [131]. While normal prostate cells do not undergo the classical oxidative phosphorylation and are programmed to undergo a particular and extremely inefficient citrate-oriented metabolism transforming glucose into citrate, then citrate is secreted as a part of the seminal liquid [132, 133]. Nevertheless, primary PCa cells turn to favor oxidative phosphorylation instead of enhancing glycolysis [133, 134]. The malignant cells are reprogrammed to oxidize citrate and complete the tricarboxylic acid cycle, thus transforming into energy-efficient malignant cells [135–137]. The consequently low level of citrate is also regarded as a potential non-invasive biomarker for PCa early diagnosis [138]. Interestingly, this alteration is just an early-stage event during the malignant progression. When PCa cells develop into metastatic or castration-resistant stages, they begin to exert the Warburg effect and have a high glucose consumption [139, 140]. Notably, the low level glycolysis may be the underlying reason why even the leading-edge instrument like FDG-PET should omit the hidden lesions of PCa in the early stage [140, 141].

It was widely hypothesized that melatonin enters freely into human cells via passive diffusion across the cellular lipid bilayer due to its amphiphilic nature [142, 143]. However, even though many reports have demonstrated that melatonin has a direct function in inhibiting tumor proliferation, its concentration does not equilibrate outside and inside cells. So there exists an underlying facilitated diffusion or an active process rather than simple passive diffusion. A series of studies by Hevia et al. explained the detailed mechanism. Firstly, they found blocking protein synthesis could unexpectedly inhibit melatonin uptake in PCa cells but extracellular Ca^2+^/K^+^ alterations failed to modify the profile of melatonin uptake [144]. Furthermore, they confirmed the role of GLUT1 in transporting melatonin into PCa cells [145]. Docking simulation assays revealed that melatonin and glucose share the same binding sites in GLUT1. Melatonin can suppress the uptake of glucose through competitive inhibition and regulates GLUT1 gene expression in PCa cells. In vivo study also confirm that glucose supplementation accelerated PCa growth in TRAMP mice while adding melatonin to drinking water reversed glucose-triggered tumor growth and expanded the lifespan of tumor-bearing mice. Previous studies also demonstrated that GLUT1 is overexpressed in PCa cells and is highly correlated with cancer proliferation and tumor malignancy [146–149].

**Fig. 3:**
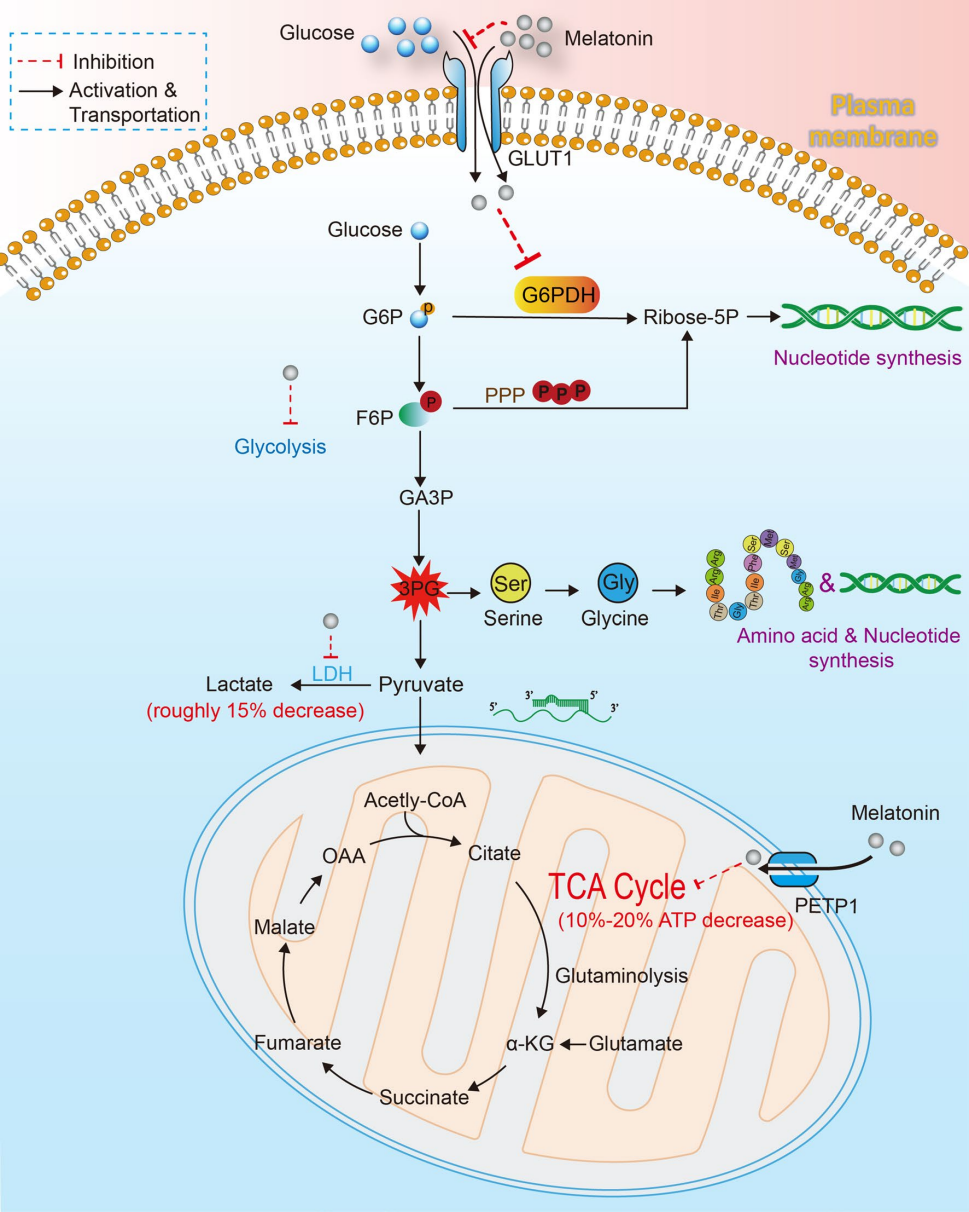
Inhibition of melatonin on PCa metabolism. Melatonin can be actively transported into PCa cells via GLUT1 and thus suppressing the uptake of glucose via competing for the binding sites of GLUT1. PEPT1/2 embedded in mitochondrion membrane actively transported melatonin into mitochondrion. Melatonin can cause 10–20% decrease in ATP production via limiting the TCA cycle and cause roughly 15% decrease of lactate via inhibiting glycolysis

Mitochondrion is regarded as the “energy factory” inside cells where the oxidative phosphorylation and electron transport chain (ETC) proceed. At the same time while ATP is being produced, some unwanted by-products, such as reactive oxygen species (ROS) and reactive nitrogen species (RNS), are also released. The high affinity to mitochondrion renders mitochondrion a biological target of melatonin [24, 150]. As Huo et al. [151] reported, the transmembrane transportation of melatonin into mitochondrial promoted its oncostatic effect on human PCa cells. PEPT1/2 embedded in mitochondrion membrane actively transported melatonin into mitochondrion and consequently induced apoptotic pathway. Hevia et al. [152] demonstrated melatonin can limit glycolysis as well as the tricarboxylic acid (TCA) cycle and pentose phosphate pathway in PCa cells. By conducting a ^13^C stable isotope-resolved metabolomic study, they found melatonin could significantly decrease glucose uptake, ATP production, LDH activity and almost all of the intermediates of the TCA cycle. The results implied a general negative effect of melatonin on glucose uptake and utilization in PCa cells (Fig. 3). Dauchy et al. [59] showed that mice bred in blue-tinted rodent cages have an elevated melatonin level in circulation, which inhibited the metabolism rate and growth of PCa xenografts.

### PCa angiogenesis and neuroendocrine differentiation

Angiogenesis is an essential physiological process that occurs in tissue healing and embryonic development. Cancerous neovascular vessels build a bridge between tumor tissues and the pre-existing tissues. The newly sprouted vessels help exchange wastes and nutrients between tumor and human body and provide a new route for tumor cells to migrate and renew [36, 153]. Thus, angiogenesis is critical for tumor progression [154] and is regarded as a new hallmark of cancers [155]. For PCa, although there are no convincing markers to evaluate the angiogenic rate, intratumoral microvessel density (MVD) is recognized as a good biomarker. Detection of contrast-enhanced ultrasonography also confirmed a higher signal intensity of blood flow in advanced PCa patients [156]. Patients with higher MVD tended to show higher Gleason scores and worse prognosis [157, 158].

The initiation of angiogenesis can be seen as an imbalance between angiogenesis inducing factors and angiogenesis inhibitory factors that favor the former group. Vascular endothelial growth factor (VEGF), an extensively studied inducer, is confirmed to be associated with PCa angiogenesis. Previous studies showed that the expression level of VEGF-A, a VEGF isoform, is up-regulated in PCa and is correlated with distant metastasis and overall prognosis [159, 160]. It is noteworthy that androgen and AR participate in angiogenesis partially via regulating VEGF and its upstream regulators [161]. For instance, HIF-1α, a well-established modulator of VEGF, is demonstrated to be activated by DHT [162, 163]. Interestingly, castration treatment can decrease the oxygen content in the microenvironment [164, 165]. While the low-oxygen environment activates HIF-1α to enhance the transcription of AR in PCa cells [166].

A preliminary clinical study demonstrated that patients who accepted melatonin treatment showed decreased serum levels of VEGF, showing the potential anti-angiogenic activity of melatonin [167]. Several studies have also pointed out that melatonin can inhibit tumor growth by blocking the development of neovascularization [167–170].

The pharmacologic concentration of melatonin (1 mM) is confirmed to inhibit the translation of HIF-1α protein while not decreasing its protein stability or mRNA transcription and the inhibition of HIF-1α consequently leading to the down-expression of VEGF-A in PCa cells [171]. Interestingly, the physiological concentration of melatonin (1 nM) fails to show the identical ability compared to the high concentration. Cho et al. [172] further reported an SHPK1-mediated regulation of melatonin on HIF-1α. Sphingosine kinase 1 (SHPK1) is a known HIF-1α regulator via maintaining HIF-1α stability [173] and melatonin at 1 mm dramatically reduced SPHK1 expression as well as HIF-1α and VEGF in hypoxic PC-3 cells. MicroRNA is a kind of noncoding RNA containing about 22 nucleotides. MicroRNA inhibited gene expression through sequence-specific interaction with the untranslated region (UTR) of homologous mRNA [174, 175] and melatonin was proved to regulate microRNA to inhibit tumor angiogenesis. Sohn et al. [176] reported that treating hypoxic PC-3 cells with melatonin could up-regulate the expression of miRNA3195 and miRNA374b. MiRNA3195 and miRNA374b, in turn, restrain the level of HIF-1α and VEGF at a transcriptional level and thus inhibited the angiogenesis and migration abilities of PCa cells.

Neuroendocrine differentiation (NED) is a commonly observed phenotypic change during ADT. NED cells are characterized by the expression of NE markers, including NSE, CgA, gastrin, and neurotensin [177]. This phenotype change is believed to be correlated with tumor progression, poor prognosis, and hormone-refractory. Typically, three main methods are leading to the NED [178], (1) Androgen depletion-induced NED [179, 180], (2) cAMP-induced NED [181, 182], (3) Cytokines-induced NED [183, 184]. Other agents such as HB-EGF [185] or Vasoactive intestinal peptide (VIP) [186] are also reported to mediate the progression of NED.

Interestingly, although NED seems to be a negative factor of PCa, melatonin still can induce this transformation without damaging its anti-proliferation effects [187]. Sainz et al. [188] reported that after being cultured with melatonin for six days, cells began to exhibit the NED characteristics and express a high level of NSE via an MT1-independent and PKA-independent pathway since the elevation of the cAMP level is transient. Mayo et al. [189] demonstrated a further underlying mechanism that involves activated MAPK/ERK 1/2 pathway, redox regulation and androgen receptor nuclear exclusion. Melatonin treatment not only increased the intracellular GSH level but also enhancing the ADT-dependent NED. And genomic microarray showed that IGFBP3 is the key gene that regulates the NED process of melatonin. In addition, Rodriguez-Garcia et al. [190] confirmed that NED does not increase survival chances for PCa cells. On the contrary, melatonin-induced NED may be responsible for greater sensitivity to cytokines, namely TNFα and TRAIL.

According to Wang et al. [191], there is a potential correlation between PCa angiogenesis and NED. Previous studies pointed out that PCa specimens with a high degree of NED also have more neovascularization and VEGF staining [192]. Moreover, although being reported in separate diseases or biological contexts, proteins, such as CHGA, p53 and HIF-1α, that regulate angiogenesis also participate in the progress of NED [191]. While as we have discussed that melatonin can inhibit angiogenesis and promote NED, there seems to be a contradiction in the effect of melatonin on PCa. Thus, we speculate that the transformation of NED is an incidental effect of melatonin on increasing cell sensitivity to cytokines treatment, and the inhibitory effect on angiogenesis is the more important one. Of course, more specific mechanisms of this contradiction remain to be studied.

### Melatonin and apoptosis of prostate cancer cells

Apoptosis, a genetically programmed cell death, is highly conserved among many species [193]. Moderately activated apoptosis helps to clear unwanted cells such as dead white blood cells in immune responses or cells with harmful mutations caused by external stimuli [194, 195]. The balance between cell growth, dormancy, apoptosis is of great importance to maintain homeostasis while dysregulation of this harmonious relationship is an underlying mechanism of tumorigenesis and is also regarded as a hallmark of cancers [155, 196, 197]. The well-accepted apoptotic features include cell contraction, chromatin aggregation, and DNA ladder formation caused by internucleosomal DNA fragmentation, which ends with phagocytosis by macrophages or adjacent cells, thus avoiding inflammatory reactions among surrounding tissues [198].

As a nature oncostatic hormone, the ability of melatonin to promote cell death is validated in many types of cancers [199], and similar outcomes are also found in PCa. Joo et al. [200] showed that treating the androgen-sensitive LNCaP cells with melatonin clearly increased the number of apoptotic cells with a significant up-regulation of apoptosis-related proteins Bax and Cyt c and a decrease of survival protein Bcl-2, via activating the JNK and p38 cascade. Sainz et al. [201] examined the effect of melatonin when given in combination with TNFα or γ-radiation. Results showed that melatonin obviously arrests PCa cells in the G0/G1 phase with an increase of p21 protein and significantly elevates the efficiency of TNFα treatment via inactivating NF-κB. However, melatonin failed to enhance the apoptosis induced by γ-radiation due to the increment of intracellular glutathione. Rodriguez-Garcia et al. [190] further investigated whether melatonin could promote drug-induced apoptosis combined with doxorubicin, docetaxel, and etoposide or cytokines like TNFα and TRAIL. Interestingly, results showed that melatonin exclusively promoted cell toxicity caused by cytokines but did not appear to promote the efficiency of other chemotherapeutic drugs.

### The synergistic interactions of melatonin and other drugs

Melatonin is an endogenous oncostatic agent that displays almost null toxicity to human body [202, 203]. The synergistic interactions of melatonin and other drugs are found to achieve an ideal therapeutic effect and reduce side-effects [204–206]. The similar anti-proliferative effect of melatonin and other anticarcinogens present on human tumors in vivo and in *vitro* abstracts researchers to make further investigation.

As Reiter et al. reported, proper use of melatonin combined with other oncostatic agents can enhance the therapeutic effect [207]. For instance, DHA, a fatty acid present in the human diet, can exert a pro-apoptotic effect against PCa cells via Akt-mTOR signaling. In an in vitro study, Tamarindo et al. [208] found that melatonin combined with DHA could suppress proliferative prostate diseases through modulating mitochondrion bioenergy via AKT and ERK1/2 pathway. In another animal model research, Terraneo et al. [209] reported that they developed a noninvasive and painless therapy for PCa which combined melatonin and cryopass-laser treatment. Via cryopass-laser, melatonin could be precisely delivered to specific areas avoiding false distribution in non-target tissues and unwanted side-effects. 3 mg/kg/week melatonin (0.09 mg/mouse/week) delivered by i.p. injections could effectively inhibit the proliferation of LNCaP PCa cells. This study brought a bright future for devising alternative ways to deliver melatonin in clinical contexts.

## Conclusion

Prostate cancer (PCa) is one of the most common cancers among male patients. In 2020, nearly 191,930 new cases and 33,330 deaths of PCa are estimated with a constantly rising trend. Melatonin (N-acetyl-methoxy-tryptamine), an indole-like neurohormone, is synthesized and secreted from the pineal gland. Melatonin is mainly produced in a dark condition while light could inhibit the internal synthesis. Melatonin could exert multiple biological effects, especially as a free radical scavenger. Recently, melatonin emerges as a prospective therapeutic agent with a series of beneficial effects on human cancers over a wide range of concentrations and tumor types. In this review article, we predominantly discuss the anti-tumor effects of melatonin on human PCa. The underlying mechanisms of how melatonin inhibits the growth and progression of PCa were related to the promotion of AR exclusion, activation of MT1 signaling, modulation of PCa metabolism, inhibition of angiogenesis, regulation of neuroendocrine differentiation, induction of apoptosis. Moreover, melatonin also exerts synergistic benefits while incorporating with other chemotherapeutic agents and is an effective adjuvant to androgen depletion therapy. Melatonin is also hopeful to be a noninvasive biomarker for predicting PCa since a low urinary melatonin level is highly correlated to the incidence of PCa. Although substantial evidence suggests that melatonin could be a novel strategy for the treatment of PCa, compared with the well-explored relationship between melatonin and breast cancer, further clarification of the melatonin-mediated inhibitory effects on PCa deserves more attention. It is clear that several unsolved issues need to be resolved. For instance, only a few surveys were conducted in a clinical situation while the majority of the above findings remain in the phase of in vivo and in vitro experiments. More efforts need to be spared to translate currently non-clinical trials of melatonin to clinic use. All in all, we hope data compiled in this review will help promote the utilization of melatonin in overcoming human prostate cancer.

## Data Availability

Not applicable.

## Abbreviations

aMT6s: 6-Sulfatoxymelatonin
ADT: Androgen depletion therapy
ALAN: Artificial light at night
AR: Androgen receptor
BPH: Benign prostatic hyperplasia
CRPC: Castration resistant prostate cancer
DAG: Diacylglycerol
DHA: Docosahexaenoic acid
DHT: Dihydrotestosterone
FDG-PET: Fluorodeoxyglucose positron emission tomography
IP3: Inositoltriphosphate
PCa: Prostate cancer
PKA: Protein kinase A
PKC: Protein kinase C
PLC: Phospholipase C
PLD: Phospholipase D
PSA: Prostate specific antigens
RGS: Regulators of G-protein signaling
RNS: Reactive nitrogen species
ROR: Retinoic acid-related orphan receptor
ROS: Reactive oxygen species
RZR: Retinoid Z receptor
TRAMP: Transgenic adenocarcinoma of a mouse prostate
